# Surface melting of a colloidal glass

**DOI:** 10.1038/s41467-022-34317-2

**Published:** 2022-11-03

**Authors:** Li Tian, Clemens Bechinger

**Affiliations:** Fachbereich Physik, Universität Konstanz, 78464 Konstanz, Germany

**Keywords:** Glasses, Statistical physics, Materials science

## Abstract

Despite their technological relevance, a full microscopic understanding of glasses is still lacking. This applies even more to their surfaces whose properties largely differ from that of the bulk material. Here, we experimentally investigate the surface of a two-dimensional glass as a function of the effective temperature. To yield a free surface, we use an attractive colloidal suspension of micron-sized particles interacting via tunable critical Casimir forces. Similar to crystals, we observe surface melting of the glass, i.e., the formation of a liquid film at the surface well below the glass temperature. Underneath, however, we find an unexpected region with bulk density but much faster particle dynamics. It results from connected clusters of highly mobile particles which are formed near the surface and deeply percolate into the underlying material. Because its thickness can reach several tens of particle diameters, this layer may elucidate the poorly understood properties of thin glassy films which find use in many technical applications.

## Abstract (web summary)

The melting process in glasses is not fully understood. Experiments with colloidal glasses now show that during melting, a liquid film develops at the surface, below which a region forms with highly mobile particles. This surface glassy layer reflects the properties of the surface and the underlying bulk material.

## Introduction

Solids typically begin to melt far below their bulk melting temperature by the formation of a liquid layer at their surface^1,2^. Such surface melting which originally has been observed by Faraday in 1842 in noting a quasi-liquid layer on ice has been reported for many crystalline materials^2–5^. Unlike crystals where the presence of a fluid on top of an ordered solid is detected, e.g., by neutron or X-ray scattering experiments^1,2,6^, the demonstration of surface melting in glasses is more challenging due to the lack of appropriate order parameters distinguishing a glass from a liquid^7–12^. Although the transition of a liquid into a glass qualitatively differs from how a liquid turns into a crystal, surface melting is also predicted for amorphous materials^13–16^. Apart from basic scientific interest, surface melting of glassy systems is expected not only to influence its surface properties but may also explain the unusual behavior of thin polymeric and metallic glassy films whose reduced glass-transition temperature and strongly enhanced surface mobility is exploited in technical applications^17–22^. Despite considerable effort, however, the microscopic changes taking place near a glass surface during surface melting have not yet been resolved.

Here, we present real-space experiments of the surface melting of a two-dimensional (2D) colloidal glass where the motion of particles is fully resolved in space and time. We find that the glass melts starting from the surface by forming a broad transient region composed of a liquid and supercooled liquid in coexistence with an underlying bulk glass (BG). Surprisingly, adjacent to the BG, we observe a region with bulk density but a faster particle dynamics, the latter resulting from connected cooperative clusters of highly mobile particles which are formed at the surface and proliferate deep into the system. The thickness of this unexpected region varies non-monotonically with the effective temperature and becomes largest near the bulk glass transition point.

## Results

**Fig. 1:**
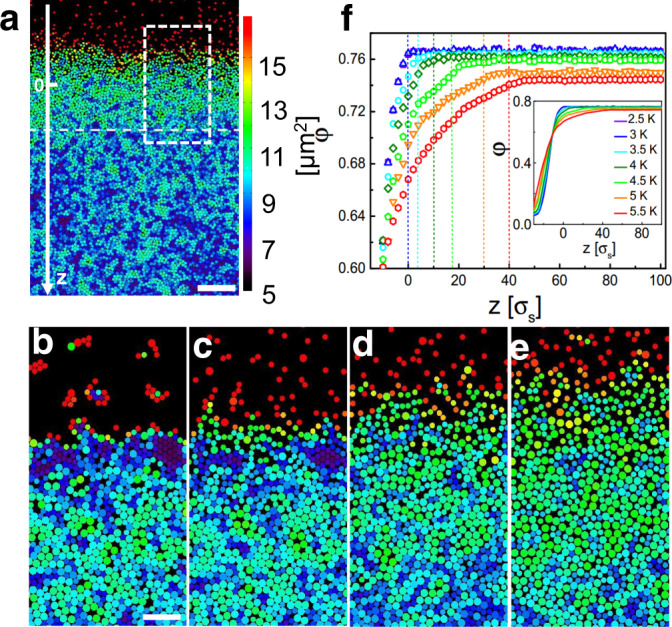
Surface melting of an attractive colloidal glass. **a** Snapshots of a glass surface at temperatures Δ*T* = 4.5 K. The color code represents the local Voronoi area. The dashed horizontal line indicates the location of ${z}_{{{{{{{{\rm{sat}}}}}}}}}^{\varphi }$ as defined in (**f**). The scale bar is 50 *μ*m. The origin of the z-axis has been defined by ${z}_{{{{{{{{\rm{sat}}}}}}}}}^{\varphi }$ for 4.5 K. **b**–**e** Typical zoom-in snapshots of the glass surface area (see dashed rectangular area in (**a**)) at different temperatures: Δ*T* = 2.5 K (**b**), 3.5 K (**c**), 4.5 K (**d**), 5.5 K (**e**). The scale bar is 15 *μ*m. **f** Depth-resolved particle area fraction for different Δ*T* near the saturation and over the entire *z* range (inset). Dashed lines indicate ${z}_{{{{{{{{\rm{sat}}}}}}}}}^{\varphi }(\Delta T)$ where the corresponding area fractions reach 99.5% of the corresponding *φ*_sat_. To compare profiles with different temperatures, ${z}_{{{{{{{{\rm{sat}}}}}}}}}^{\varphi }(\Delta T=2.5\,{{{{{{{\rm{K}}}}}}}})$ was chosen as the origin of the *z*-axis.

To yield a quasi-equilibrated gas-solid interface in a 2D colloidal system, an attractive particle interaction is required. In our experiments this is achieved by critical Casimir forces which arise due to fluctuations of the solvent’s composition near its critical temperature *T*_*c*_^23^. Upon variations of the temperature Δ*T* = *T*_*c*_−*T*, one can control the attraction between colloids suspended in the critical mixture in a fully reversible manner^24^. Note that higher Δ*T* corresponds to a weaker attraction strength in our system, yielding a higher effective temperature. The solvent is an aqueous micellar solution of non-ionic surfactant C_12_E_5_ with a lower critical point at *T*_*c*_ ≈ 32 °C and 1.2% surfactant weight^25,26^. A binary mixture of silica particles (ratio 0.55:0.45) with diameters *σ*_*s*_ = 2.4 *μ*m and *σ*_*l*_ = 3.34 *μ*m was added to the solvent which was contained in a sample cell with 100 *μ*m in height. Due to gravity the particles sediment towards the bottom of the cell where they form a disordered monolayer. The Debye screening length of the system is about 30 nm ^26^, leading to rather short-ranged particle repulsion. To create a free surface between a low density gaseous and a high density glass phase, the sample cell was first tilted by 1.15° leading to a lateral density gradient across the sample. During this step the temperature was kept at Δ*T* = 11 K where critical Casimir forces are negligible (Supplementary Fig. 1). Afterwards the sample was aligned horizontally with the temperature slowly (0.2 K/h) increased to Δ*T* = 2.5 K. As a result, a free and equilibrated surface perpendicular to the original tilting direction develops (Fig. 1). Starting from such conditions, we slowly varied the temperature to yield thermally equilibrated states at different Δ*T*. Prior to each measurement, samples were kept at the corresponding temperature for at least three hours. For further details regarding the sample equilibration, we refer to Supplementary Note 2.

Figure 1 a shows a typical snapshot for Δ*T* = 4.5 K following the above protocol. The particles are colored according to their Voronoi cell area, highlighting their local area fraction *φ*. From the top to the bottom (i.e. in the direction of the *z*-axis) we observe a smooth transition from a highly diluted gas phase (red) to a densely packed (blue) disordered state. Figure 1b–e shows enlarged snapshots of the dashed region in Fig. 1a for Δ*T* between 2.5 and 5.5 K (Supplementary Video 1). Due to the temperature-dependent critical Casimir attraction, the interface becomes increasingly broadened with increasing Δ*T* which hallmarks the surface melting (see area fraction profiles in Fig. 1f). In contrast to the strong temperature dependence near the surface, the profiles almost perfectly overlap at large *z* where they converge to *φ*_sat_ which only slightly (<5%) varies with Δ*T*. The depths ${z}_{{{{{{{{\rm{sat}}}}}}}}}^{\varphi }(\Delta T)$ where the profiles saturate are shown as vertical dashed lines in Fig. 1f. Since the properties of glasses strongly depend on their density, we have also determined the variance of the z-resolved local area fraction. Both quantities saturate at the same depth which confirms that ${z}_{{{{{{{{\rm{sat}}}}}}}}}^{\varphi }$ is a reliable measure for the depth below which the structural properties of the system become constant (Supplementary Note 3). Notably, we find a rather continuous increase of *φ* between the gas and the liquid, which is due to the averaging parallel to the rough surface of the disordered system.

**Fig. 2:**
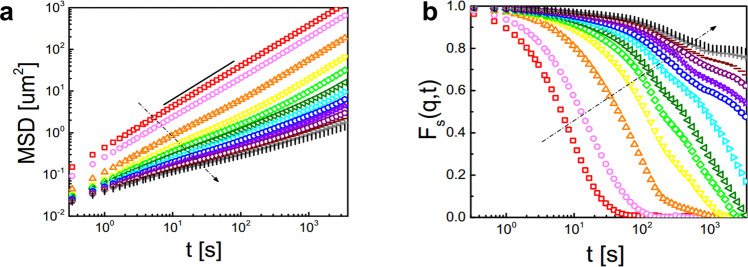
Depth-resolved dynamical properties. **a** Mean squared displacement (MSD) and (**b**) intermediate scattering function *F*_*s*_(*q*, *t*) for different *z* values (in units of *σ*_*s*_): −25, −15, −5, 5, 15, 25, 35, 45, 55, 65, 75, 85, 95 (in the direction of the arrow) where *q* = 1.45 *μ*m^−1^ corresponds to the first peak in the structure factor at *φ*_sat_. Each bin is averaged over a width of *z* ± 5*σ*_*s*_. The data is taken at Δ*T* = 4.5 K. The black solid line corresponds to the slope equal to 1.

To investigate how the material’s properties change with increasing distance to the surface, we evaluated the *z*-resolved mean-squared displacement (MSD) and intermediate scattering function *F*_*s*_(*q*, *t*) (Supplementary Note 3). Exemplarily, this is shown for Δ*T* = 4.5 K in Fig. 2 but the same qualitative behavior is also found for the other temperatures considered in this work. Near the surface (*φ* < 0.2, particles shown in red in Fig. 1a) the dynamics is diffusive with the diffusion coefficient being almost identical to that of isolated particles. We note that small deviations from a strictly linear MSD are observed at larger times due to the attractive particle interaction which can lead to the temporary formation of small particle clusters. We refer to this region as a gas phase. With increasing *z*, the dynamics first remains diffusive but with a gradually decreasing diffusion coefficient (Fig. 2a). In this range, the corresponding *F*_*s*_(*q*, *t*) rapidly decays to zero (Fig. 2b) suggesting a liquid layer. At even larger depths (i.e. larger *φ*) particle displacements require increasingly cooperative rearrangements, leading to a sub-diffusive behavior^27^. Such cooperative behavior is supported by the strong increase of the decay time of *F*_*s*_(*q*, *t*) (supercooled liquid). At depths *z* > 35*σ*_*s*_ the MSD and *F*_*s*_(*q*, *t*) exhibit a plateau-like structure which is characteristic when the dynamics of particles is dominated by the cages formed by their neighbors as being characteristic for bulk glasses^28^.

Since the particle area fraction gradually increases from the surface towards the bulk, the observation of a smooth transition (liquid - supercooled liquid–glass) may simply reflect the density dependence of the phase behavior of a disordered colloidal system. This, however, is not in agreement with our results. Opposed to the area fraction which saturates for Δ*T* = 4.5 K at *z* ≈ 18*σ*_*s*_ (Fig. 1f), pronounced variations in *F*_*s*_(*q*, *t*) are clearly visible even below *z* ≈ 65*σ*_*s*_ (Fig. 2b). Such decoupling of the intermediate scattering function from the area fraction is not observed in bulk glasses and must therefore originate from the presence of the surface. We note, that a decoupling of static and dynamic properties at different depths has been also reported in numerical simulations of thin glassy films of attractive polymers^29^.

Because *F*_*s*_(*q*, *t*) decays rather slowly at large depths, a quantitative analysis of its characteristic decay time is difficult on our experimental time scales. Therefore, we have also calculated the self-part of the overlap function *q*_*s*_(*t*, *z*) which measures how similar particle configurations remain after time *t* over distance *z*^30–32^. This quantity displays a similar behavior as *F*_*s*_(*q*, *t*) but decays considerably faster. For a definition of the self-part of the overlap function we refer to Supplementary Note 3.

**Fig. 3:**
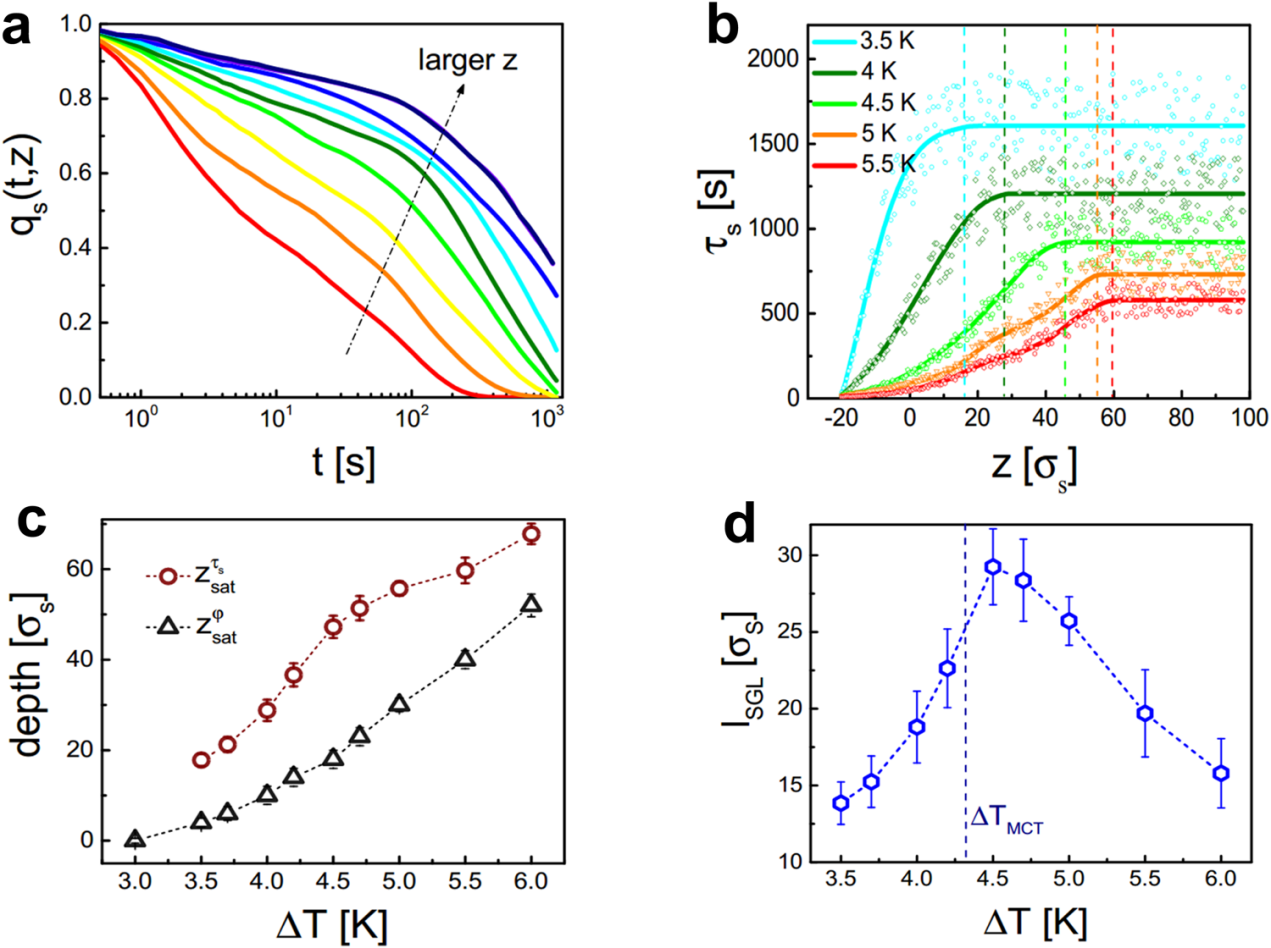
Overlap function and surface glassy layer. **a** Time-dependence of the overlap function for Δ*T* = 4.5 K for the following *z* values (in units of *σ*_*s*_ and in the direction of the arrow): −20, −15, −5, 0, 10, 20, 30, 45, 80. **b** Measured (symbols) and averaged (lines) values of the depth-dependent relaxation time *τ*_*s*_(*z*) for different temperatures. The solid lines correspond to smoothed experimental data obtained from averaging over 10 data points, each. Vertical dashed lines denote ${z}_{{{{{{\rm{sat}}}}}}}^{{\tau }_{s}}$ where *τ*_*s*_(*z*) saturates. **c** Temperature-dependence of ${z}_{{{{{{{{\rm{sat}}}}}}}}}^{\varphi }$ and ${z}_{{{{{{\rm{sat}}}}}}}^{{\tau }_{s}}$ with a surface glassy layer (SGL) in between. For $z\ge {z}_{{{{{{\rm{sat}}}}}}}^{{\tau }_{s}}$ we observe a bulk glass (BG) while for $z\le {z}_{{{{{{{{\rm{sat}}}}}}}}}^{\varphi }$ a gas/fluid state is found. Error bars correspond to the standard deviation of the corresponding mean values. **d** Thickness *l*_SGL_ of the surface glassy layer as a function of the temperature with the transition point according to mode coupling theory shown as a vertical line. The error bars correspond to the total error due to the error bars shown in **c**.

Figure 3a shows the temporal decay of *q*_*s*_(*t*) for increasing depth at a temperature Δ*T* = 4.5 K (qualitative similar results are observed over the entire temperature range considered in this work). We define the corresponding relaxation times *τ*_*s*_(*z*) as the time to reach *q*_*s*_(*t*) = 0.4. As seen in Fig. 3b, *τ*_*s*_(*z*) increases with *z* and eventually saturates at the temperature-dependent depth ${z}_{{{{{{\rm{sat}}}}}}}^{{\tau }_{s}}(\Delta T)$ which marks the transition towards the bulk glass. Similar to *F*_*s*_(*q*, *t*), *τ*_*s*_(*z*) only saturates considerably below the depth ${z}_{{{{{{{{\rm{sat}}}}}}}}}^{\varphi }$ where the area fraction becomes constant (Fig. 3c). In the following we are referring to the region ${z}_{{{{{{{{\rm{sat}}}}}}}}}^{\varphi }\le z\le {z}_{{{{{{{{\rm{sat}}}}}}}}}^{\tau }$ as a surface glassy layer (SGL). Remarkably, the thickness of the SGL, i.e., ${l}_{{{{{{{{\rm{SGL}}}}}}}}}={z}_{{{{{{{{\rm{sat}}}}}}}}}^{\varphi }-{z}_{{{{{{{{\rm{sat}}}}}}}}}^{\tau }$ varies non-monotonically as a function of the temperature with a maximum at Δ*T* ≈ 4.5 K (Fig. 3d). This maximum is found to be close to the bulk glass transition point (dashed vertical line in Fig. 3d and Supplementary Fig. 4d).

**Fig. 4:**
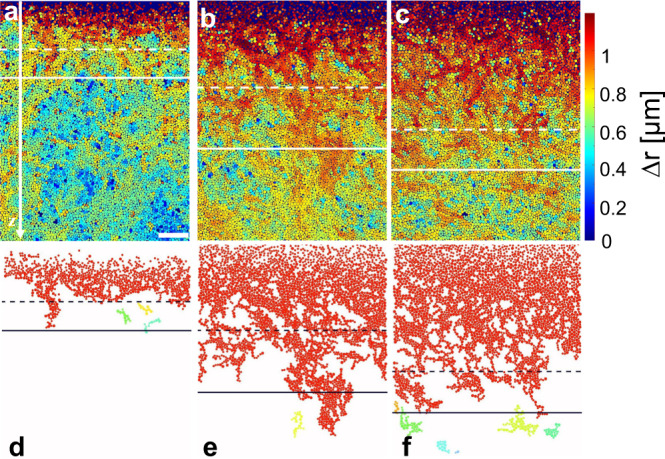
Depth-dependent particle displacements. **a**–**c** Typical snapshots with particles colored according to their displacements within 333 s (being much larger than the beta relaxation time *τ*_*β*_ ( < 30 s) for three different temperatures (from left to right Δ*T* = 3.5 K (**a**), 4.5 K (**b**), 5.5 K (**c**)). The horizontal dashed and solid horizontal lines correspond to ${z}_{{{{{{{{\rm{sat}}}}}}}}}^{\varphi }$ and ${z}_{{{{{{\rm{sat}}}}}}}^{{\tau }_{s}}$, respectively. The scale bar is 35 *μ*m. **d**–**f** Morphology of clusters comprised of connected particles whose displacement is larger than 0.3*σ*_*s*_ for Δ*T* = 3.5 K (**d**), 4.5 K (**e**), 5.5 K (**f**). Different (unconnected) clusters are shown in different colors. Dashed and solid horizontal lines correspond to ${z}_{{{{{{{{\rm{sat}}}}}}}}}^{\varphi }$ and ${z}_{{{{{{\rm{sat}}}}}}}^{{\tau }_{s}}$, respectively.

To provide a microscopic understanding of the SGL, we have analyzed how particles with different mobility are distributed across the sample at different temperatures. Figure 4a–c shows the result for Δ*T* = 3.5, 4.5, and 5.5 K where the particles are color-coded according to their displacement Δ*r* within 333 s (this timescale has been chosen since it is between the *β-* and the *α*-relaxation time of the bulk glass (Supplementary Note 5) and thus captures the cage-escape dynamics). As expected, highly mobile particles (red) are frequently found near the surface (gas, liquid) but they also proliferate by several tens of particle diameters below even beyond ${z}_{{{{{{{{\rm{sat}}}}}}}}}^{\varphi }$ (dashed horizontal lines) where the density area fraction saturates. To visualize the penetration depth of clusters comprised of fast particles in more detail, we highlighted in Fig. 4d–f connected clusters comprised of particles whose displacement Δ*r* is larger than 0.3*σ*_*s*_ (this displacement roughly corresponds to the cage size near the glass transition temperature (Supplementary Fig. 4b). The time-dependence of such regions is shown in the Supplementary Video 2. Notably, the penetration depth of these clusters beyond ${z}_{{{{{{{{\rm{sat}}}}}}}}}^{\varphi }$ (horizontal dashed lines) exhibits a very similar non-monotonic temperature dependence as the thickness of the SGL (see Fig. 3d) which provides an important clue to understand its unusual properties.

For a more quantitative support of a relationship between the SGL and clusters of highly mobile particles reaching down from the surface, we have analyzed the temperature-dependent distribution of the 10% fastest particles in the region $z > {z}_{{{{{{\rm{sat}}}}}}}^{\varphi }$ (Supplementary Fig. 6 and Supplementary Video 3). They show a very similar behavior as seen in Fig. 4d–f in particular with regard to the non-monotonic temperature dependence of the extension below ${z}_{{{{{{\rm{sat}}}}}}}^{\varphi }$. From the decay of the measured *z*-dependent probability distribution of 10% fastest particles, we obtained their decay length which is in remarkable agreement with *l*_SGL_ (Supplementary Fig. 6d, e). As shown in Supplementary Note 7, the 10% fastest particles form connected clusters and exhibit a cooperative dynamics, in resemblance to cooperative rearrangement regions (CRRs) in bulk glasses^33,34^. Unlike CRRs in bulk glasses whose size monotonically increases when approaching the glass transition point, however, in our experiments these clusters display a non-monotonic temperature dependence. This difference is due to the presence of the free surface which promotes the formation of fast particle clusters which then expand deep into the sample. At small Δ*T*, i.e. large particle attractions, the strongly reduced particle motility near the surface limits the formation of such clusters. On the other hand, at large Δ*T* the size of these clusters becomes smaller since dynamical correlations in glassy systems generally decrease with increasing effective temperature^35–37^. In combination, this leads to the non-monotonic dependence of *l*_SGL_ as observed in our experiments.

## Discussion

Our results demonstrate that surface melting of glasses is qualitatively different compared to crystals and leads to the formation of a surface glassy layer. This layer contains cooperative clusters of highly mobile particles which are formed at the surface and which proliferate deep into the material by several tens of particle diameters and well beyond the region where the particle density saturates. This might explain why the properties of thin glassy films considerably deviate from their corresponding bulk properties^38–40^. In addition, we found that the thickness of the surface glassy layer exhibits a non-monotonic dependence of the particle attraction, i.e. the effective temperature. Such behavior bears some interesting resemblance to recent observations of the non-monotonic properties of the dynamic correlation length *ξ*^dyn^ in atomic and colloidal glasses^30–32^, which is awaiting for further theoretical studies. Similar to *ξ*^dyn^ which is determined in the presence of a frozen interface and which characterizes the properties of the bulk glass^30^, the formation of a surface glassy layer during surface melting also reflects the properties of the underlying bulk material. Very recently, we became aware of another colloidal study which also observed that static and dynamic properties saturate at different depths near the surface of a glass^41^. Because in this study the attraction mechanism is different from ours, this strongly supports the idea the formation of surface glassy layers is expected to be a common phenomenon near the surface of glassy materials.

## Supplementary information

Supplementary Information

Description of Additional Supplementary Files

Supplementary Movie 1

Supplementary Movie 2

Supplementary Movie 3

## Data Availability

The data that support our findings of this study are available from the corresponding author upon request.
