# Supplementary material for: Surface melting of a colloidal glass
Source: Nat Commun. 2022 Nov 3;13:6605. doi: 10.1038/s41467-022-34317-2 (PMC9633806; doi:10.1038/s41467-022-34317-2)
Supplement: Supplementary file 1 — Supplementary Information [file 41467_2022_34317_MOESM1_ESM.pdf]

Supplementary Information for Surface melting of a colloidal glass

Li Tian[1], Clemens Bechinger[1,⋆]

[1]Fachbereich Physik, Universität Konstanz, 78464 Konstanz, Germany

⋆To whom correspondence should be addressed. E-mail: clemens.bechinger@uni-konstanz.de

Supplementary Note 1: Temperature-dependent critical Casimir forces

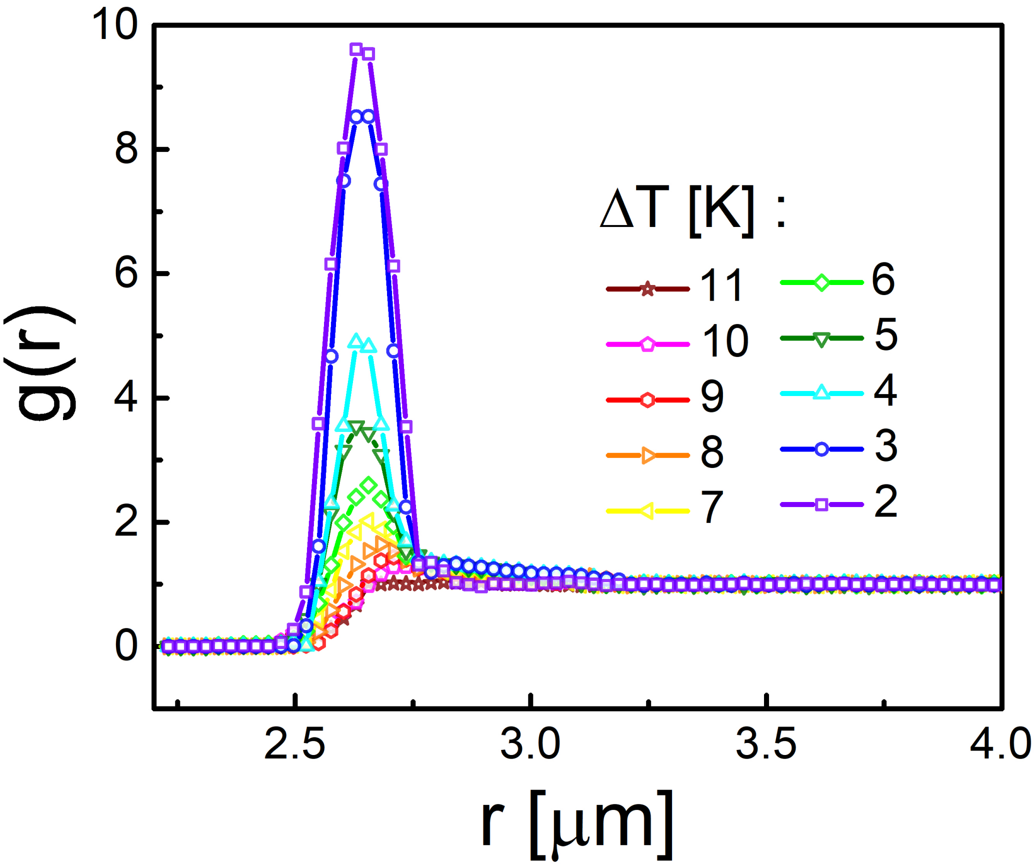

Supplementary Fig. 1 Radial distribution functions. g(r) of a dilute suspension of $\sigma = 2.4$ μm silica spheres in a critical solvent for different temperatures.

Supplementary Fig. 1 shows the temperature-dependence of the radial distribution function g(r) of a dilute sample of silica particles with diameter $\sigma = 2.4$ μm at 7% area fraction and suspended in an aqueous micellar solution of nonionic surfactant $C_{12}E_5$ at its critical

composition, corresponding to 1.2% surfactant weight [1, 2]. When approaching the critical temperature $T_c \approx 32$ °C by decreasing $\Delta T = T_c - T$, the height of the first peak in g(r) gradually increases which demonstrates an increasing particle attraction. At low temperatures, i.e. $\Delta T = 11$ K, the pair correlation function is in good agreement with a hard-sphere interaction.

Critical Casimir forces arise due to spatial confinement of concentration fluctuations of a binary mixture near its critical point. Under such conditions, the range of these fluctuations is determined by the temperature-dependent correlation length $\xi(T) = \xi_0 \mid \frac{T-T_C}{T_C} \mid^{-\nu}$ with ξ_0 a material specific value characterizing the molecular interaction range. The exponent $\nu \approx 0.63$ is a universal constant corresponding to the value of universality class of a three dimensional Ising system. In our system and for the temperatures considered here, ξ varies between 10 and 80 nm [2]. Because the correlation length is much smaller than the size of the colloidal particles in our study, critical Casimir forces do not depend on the local particle concentration but remain uniform throughout the system. Since the thickness of the sample cells used in our study is 100 µm, the fluid above the colloidal monolayer provides a huge reservoir of binary fluid. This guarantees that local (and very short-ranged) adsorption layers around the colloids do not cause concentration variations of the critical mixture on the length scale of colloids. This renders critical Casimir interactions to be independent of the local particle concentration as recently shown in experiments [2]. This further supported by experiments on the crystallization of colloidal particles in presence of critical Casimir forces which show excellent agreement with numerical simulations based on a density-independent critical Casimir interaction [3]. Notably, those simulations were performed using only pairwise interactions which suggests that many body contributions can be neglected under such conditions.

Supplementary Note 2: Sample equilibration

In order to guarantee that our system is in quasi-equilibrium during our measurements, we kept the sample for at least 3h each at the corresponding value of ΔT prior to each experiment. This timescale corresponds to more than 400 times the self-diffusion time $t_s \approx 26$ s of the particles which has been experimentally determined from the diffusion coefficient $D = 0.108$ μm^2/s. After such equilibration times the vertical density profile of our system remains - apart from small changes due to equilibrium fluctuations - constant.

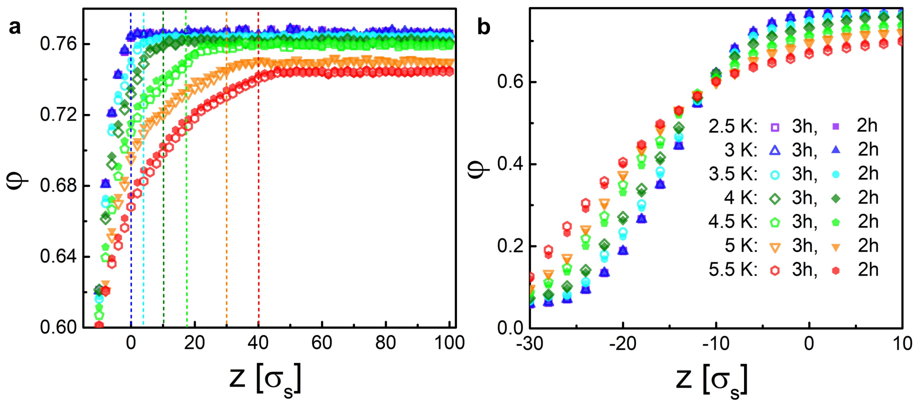

Supplementary Fig. 2 Area fraction profile for different equilibration times. Comparison of the depth-resolved area fraction after different equilibration times (2 h: closed symbols, and 3 h: open symbols). To demonstrate that both the saturation values **(a)** and the gas-liquid interfaces **(b)** are equilibrated after 2 h, we show different z-ranges of the profiles. **a**, z-ranges from $-10\ \sigma_s$ to $100\ \sigma_s$. **b**, z-ranges from $-30\ \sigma_s$ to $10\ \sigma_s$.

To demonstrate such behavior, In Supplementary Fig. 2 we compare density profiles for all temperatures presented in our work taken after 2 h and 3 h respectively (the profiles were obtained during a 10min interval at the end of the 2 h and 3 h equilibration period).

Supplementary Note 3: Depth-dependent analysis of structural and dynamic properties

To obtain z-resolved profiles of structural and dynamical properties (e.g. the area fraction profile shown in Figure 1f) we have partitioned the system into slices with thickness 2 σ_s parallel to the surface, i.e. perpendicular to the z-direction. To yield statistically reliable data, typically about 5000 frames have been evaluated. Particle positions were determined via video microscopy with a spatial resolution of about 25 nm [4]. The local area fraction has been obtained using the radical Voronoi tessellation [5, 6]. Here, the local area fraction around particle i is defined as $\varphi_i = A_i/A_{\mathrm{V}}$, where A_i is the area of particle i and A_{V} that of the corresponding Voronoi cell. The area fraction for each slice is $\varphi(z) = \frac{1}{N}\sum_{i(z)} \varphi_i$ with N the corresponding particle number within the slice.

As another confirmation that the static properties of the system are well saturated below z^{φ}_{sat}, as defined in Fig. 1, we have also calculated the depth-resolved variance $\sigma^2_{\varphi}(z)$ of the corresponding local area fraction. The variance of area fraction for each slice is $\sigma^2_{\varphi}(z) =$

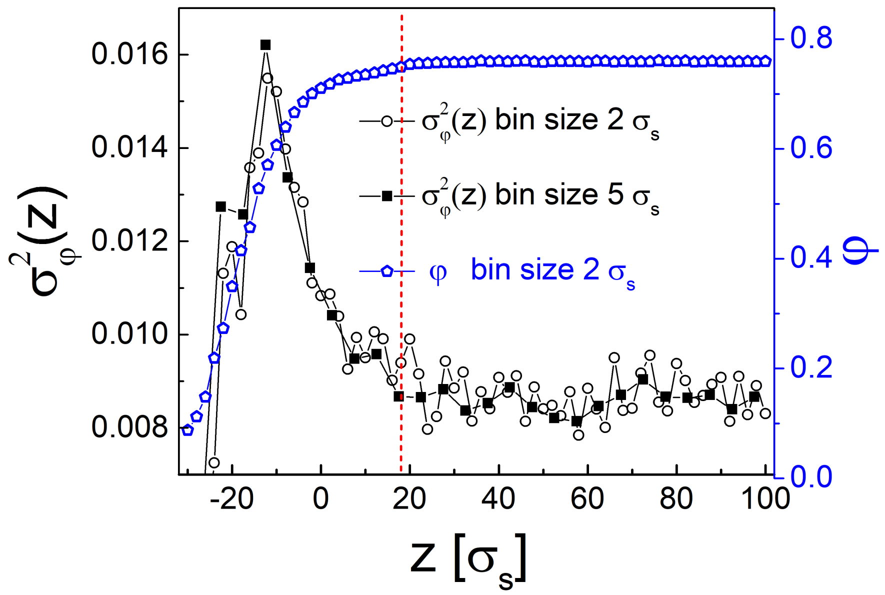

Supplementary Fig. 3 Depth-resolved mean value and variance of the particle area fraction. Measured depth-resolved variance of area fraction $\sigma^2_\varphi(z)$ for different slice widths 2 σ_s (black open circles) and 5 σ_s (close squares), respectively. For comparison we also show the mean value of area fraction φ (blue open pentagons). The vertical dashed line corresponds to z^φ_{sat}. All data are taken at $\Delta T = 4.5$ K.

$\frac{1}{N}\sum_{i(z)}(\varphi_i - \langle\varphi_i\rangle)^2$ with N the corresponding particle number within the slice. Exemplarily in Supplementary Fig. 3, we show the depth-resolved variance of particle area fraction for $\Delta T = 4.5$ K together with $\varphi(z)$. Both quantities saturate below z^φ_{sat} which corroborates our observations that the static properties of the system remain constant below this depth. The peak of the variance at $z \approx -12\ \sigma_s$ is in agreement with the position of the gas-liquid interface where the density exhibits a rather strong gradient. Similar behaviors are observed for other temperatures with the height of the peak decreasing with increasing ΔT. This is consistent with our observations that the gas-liquid interface becomes less sharp upon decreasing the particle attraction.

For extracting time-dependent z-dependent dynamical properties (MSD, $F_s(q,t)$) of our experimental data, the thickness of the horizontal slices was set to 10 σ_s. The mean square displacement (MSD) and the intermediate scattering function $F_s(q,t)$ were obtained according to MSD $=<\mathrm{r(t+t')^2 - r(t')^2}>$ and $F_s(q,t) =< \sum \exp^{(iq(r(t+t')-r(t')))} > /N$, where

the brackets correspond to an average over t', r is the particle position and N is the total number of particles. The wave vector was set to $q = 1.45\ \mu\text{m}^{-1}$, corresponding to the maximum of the static structure factor of the bulk system at large area fraction. To obtain the overlap function $q_s(t, z)$, we have divided the field of view into square boxes labelled with index j and with side length of 0.5 σ_s. Then the self-part of the overlap function is defined as $q_s(t, z) = \frac{\sum_{j(z)} < n_j^s(t+t') n_j^s(t') >}{\sum_{j(z)} < n_j^s(t') >}$, where $n_j^s(t) = 1$ if the box is occupied by the same particle at time t and $n_j^s = 0$ otherwise. The summation $j(z)$ corresponds to all particles at distance z.

Supplementary Note 4: Properties of the bulk glass

We have determined the static and dynamic properties of the bulk glass and demonstrated that our system displays the typical behaviours of a standard glass. In Supplementary Fig. 4a we show the radial distribution function g(r) within the bulk glass region, i.e. for $z > z_{sat}^{\tau_s}$. In agreement with other glass forming systems, the temperature dependence on g(r) is mild. And the corresponding inset shows that the height of the largest peak in g(r) is a smooth function of the temperature, indicating that the system is a good glass former which shows no sign of crystallization [7]. Supplementary Fig. 4b shows the time dependence of the MSD in the bulk glass region for different ΔT. As expected, the MSD exhibits a plateau-like structure which becomes increasingly pronounced upon increasing the particle attraction, i.e. when decreasing ΔT. Supplementary Fig. 4c shows the time dependence of the intermediate scattering function $F_s(q, t)$ for a wave vector corresponding to the maximum in the static structure factor, i.e. $q = 1.45\ \mu\text{m}^{-1}$. The strongly increasing decay time of $F_s(q, t)$ with decreasing ΔT is characteristic for a glass [8]. From the temperature-dependent decay of $F_s(q, t)$ we can extract the glass transition temperature. For that, the corresponding relaxation time of the bulk $\tau_\alpha^{bulk}(\Delta T)$ is defined as the time where $F_s(q, t)$ decays to $1/e$. According to mode-coupling theory (MCT), this relaxation time is related to the glass transition point ΔT_{MCT} via $\tau_\alpha^{bulk}(\Delta T) \sim (\Delta T_{MCT} - \Delta T)^{-\gamma}$ where $\gamma = \frac{1}{2a} + \frac{1}{2b} = 2.69$ has been computed using $a = 0.29$ and $b = 0.517$ obtained from $F_s(q, t)$ [9, 10]. Supplementary Fig. 4d shows that our experimental data (open symbols) are in good agreement with MCT and suggest a glass transition point near $\Delta T = 4.3$ K. Note that the area fraction is only slightly changing with temperature (Supplementary Fig. 4d, inset).

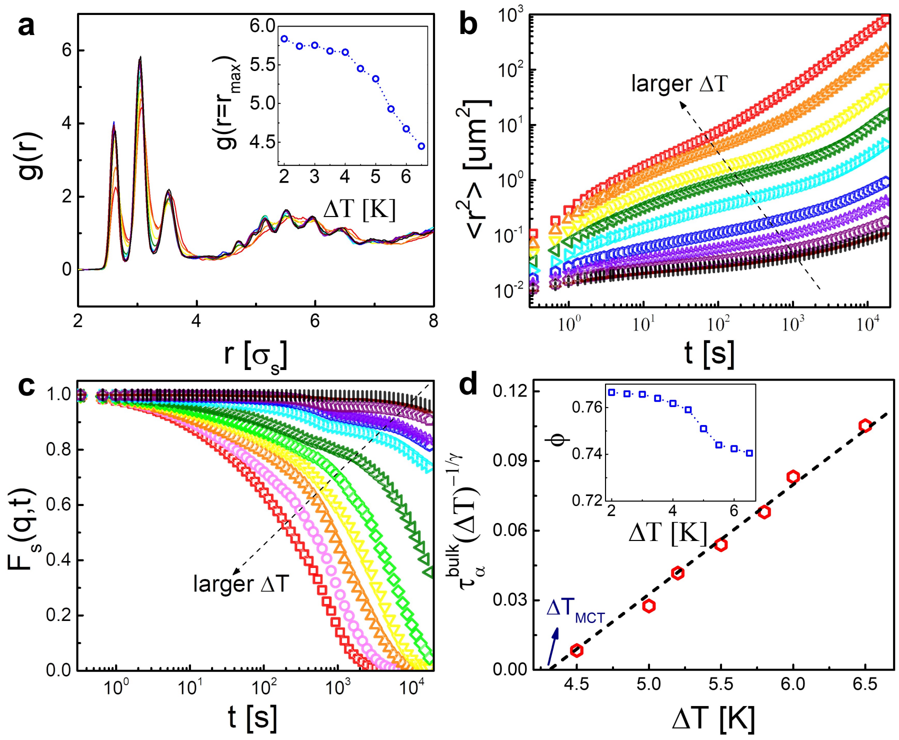

Supplementary Fig. 4 Characterization of the bulk glass. **a** T-dependent radial distribution function g(r). Inset: ΔT- dependence of the height of the largest peak in g(r). **b** Mean squared displacement for different temperatures (ΔT in unit K): 2, 2.5, 3, 3.5, 4, 4.5, 5, 5.5, 6, 6.5 (in the direction of the arrow). **c** Intermediate scattering function for ΔT= 2, 2.5, 3, 3.5 ,4, 4.5, 5, 5.2, 5.5, 5.8, 6, 6.5(in the direction of the arrow). **d** Determination of the glass transition temperature by plotting $\tau_\alpha^{bulk}(\Delta T)^{-\frac{1}{\gamma}}$ vs. ΔT. From the intercept with the x-axis the glass transition point is determined to $\Delta T \approx 4.3$ K according to MCT. The dashed line corresponds $\tau_\alpha^{bulk}(\Delta T)^{-\frac{1}{\gamma}} \sim (\Delta T_{MCT} - \Delta T)$ with $\gamma = 2.69$. Inset: area fraction as function of ΔT .

Supplementary Note 5: Beta relaxation time

The beta relaxation time is estimated according to the first (logarithmic) derivative of MSD as a function of t [11]. Exemplarily this is shown for $\Delta T = 4.5$ K in Supplementary Fig. 5. The derivative of the MSD decreases at short times and then increases again at long times. The minimum in slope becomes increasingly pronounced at large depths and corresponds to a plateau in the MSD which is characteristic for caging effects. From the

position of the minimum the beta-relaxation time has been determined to about 30 s. Similar values of the beta-relaxation time (within 10%) have been also determined for the other temperatures considered in this work.

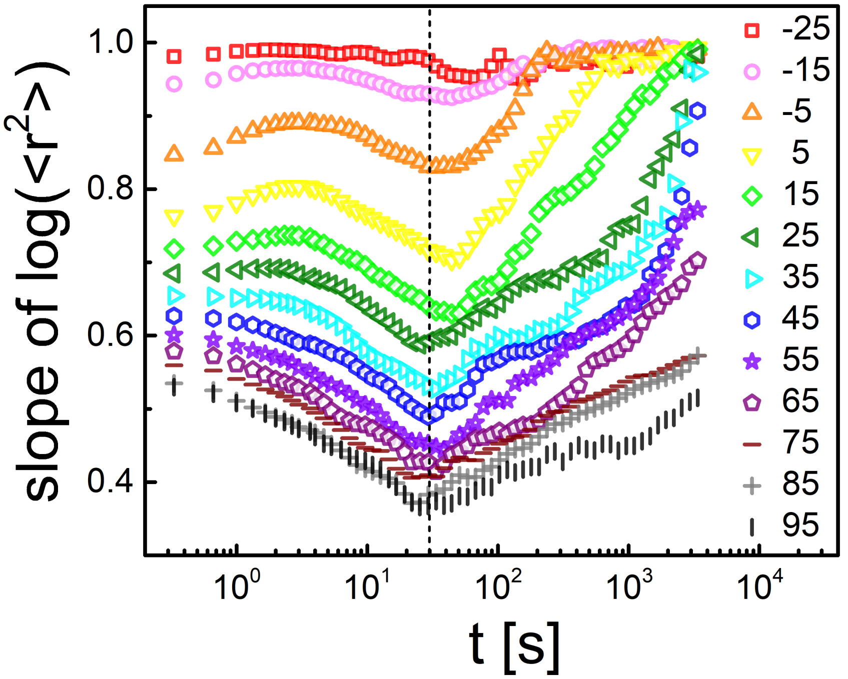

Supplementary Fig. 5 Slope of MSD. The first (logarithmic) derivative of MSD as a function of t for different depths and for a temperature $\Delta T = 4.5$ K. The time where the slope of MSD reaches a minimum at larger depths (about 30 s) corresponds to the beta relaxation time.

Supplementary Note 6: Characterization of 10% fastest particles below $z^{\varphi}_{\rm sat}$

To study how the existence of free surface influences dynamical heterogeneities below z^{φ}_{sat}, we characterized the fastest 10% within 333 s in that region. Supplementary Figs. 6a-c, shows snapshots at different temperatures with z^{φ}_{sat} and $z^{\tau_s}_{sat}$ indicated as dashed and horizontal lines. Clearly, regions of fast particles are correlated with $z^{\tau_s}_{sat}$. Supplementary Fig. 6d shows the probability distribution $p_{fast}(z)$ of fast particles for $z \geq z^{\varphi}_{sat}$ which monotonically decays and eventually saturates at depths much below z^{φ}_{sat} (similar to what is observed for $z^{\tau_s}_{sat}$). From the distance where $p_{fast}(z)$ saturates (i.e. where it approaches 98% of the corresponding saturation value), we define the depth $z_{fast}(\Delta T)$ to which fast particles

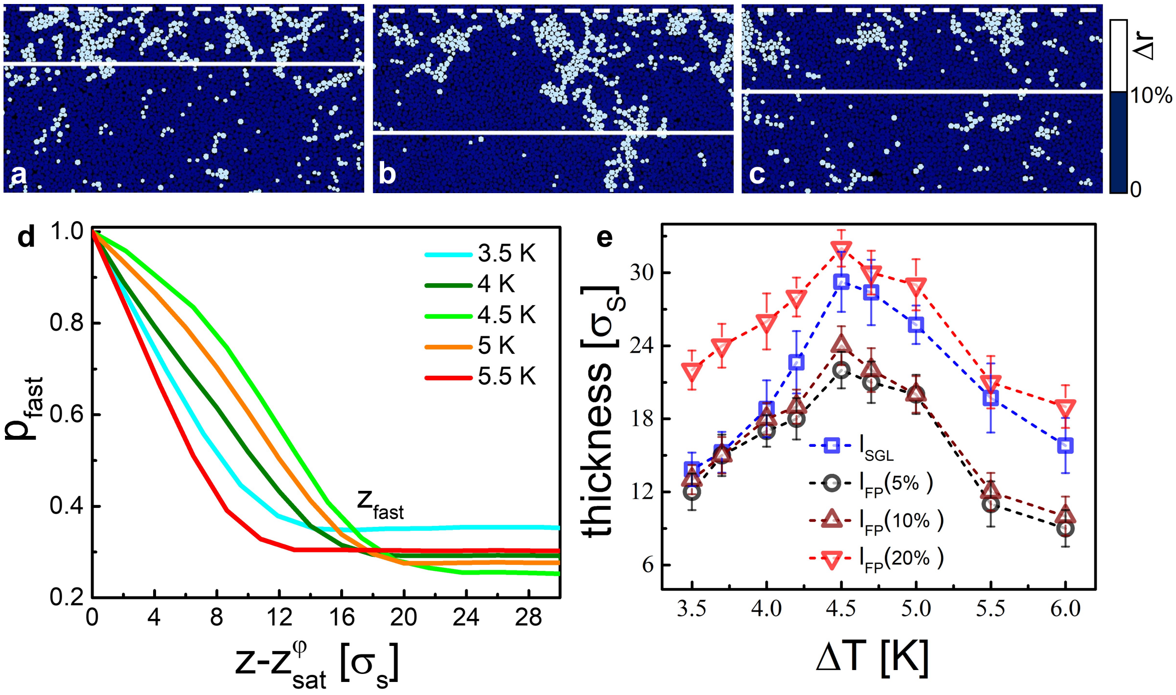

Supplementary Fig. 6 Penetration depth of 10% fast particles. a -c Fastest 10% particles (white) in the range $z \geq z^{\varphi}_{sat}$ for $\Delta T = 3.5$ K **(a)**, 4.5 K **(b)**, 5.5 K **(c)**. Dashed and solid horizontal lines correspond to z^{φ}_{sat} and $z^{\tau_s}_{sat}$, respectively. **d** Normalized probability distribution p_{fast} of fast particles. **e** Comparison of $l_{fast} = z_{fast} - z^{\varphi}_{sat}$ for 5%, 10%, 20% fast particles and $l_{\text{SGL}} = z^{\varphi}_{\text{sat}} - z^{\tau}_{\text{sat}}$, which show a very similar non-monotonic behavior versus ΔT. Error bars correspond to the standard deviation of the corresponding mean values.

penetrate. Analog to the definition of l_{SGL} we define $l_{fast} = z_{fast} - z^{\varphi}_{sat}$. Remarkably, l_{fast} displays a very similar non-monotonic dependence on ΔT as l_{SGL} (Supplementary Fig. 6e). The non-monotonic trend and in particular the position of the maximum of l_{fast} is not influenced by the threshold (percentage) value fast particles are determined, as shown in Supplementary Fig. 6e.

Supplementary Note 7: Characterization of clusters composed of the 10% fastest particles

The 10% fastest particles form clusters connected by nearest neighbours and move cooperatively within the same cluster, reminiscent the cooperative rearrangement regions (CRRs)

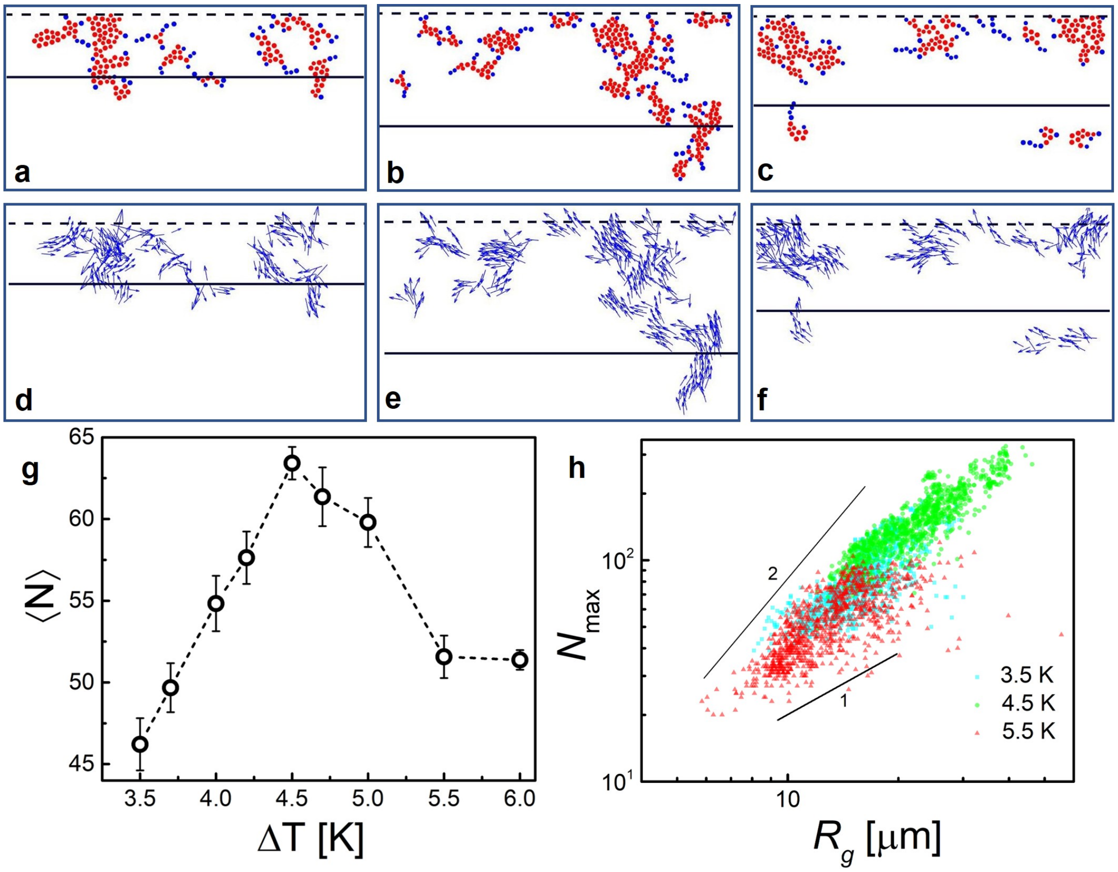

Supplementary Fig. 7 Properties of clusters comprised of fast particles. a - c Typical morphology of clusters for $\Delta T = 3.5$ K (**a**), 4.5 K (**b**), 5.5 K (**c**). The particles that have more than 2 nearest neighbors are marked red, others are marked blue. **d-f** The same clusters with individual particle displacement shown by blue arrow. The direction and length of the arrows represent the direction and magnitude of the displacement, respectively. **g** Average number of particles within clusters as a function of ΔT. Error bars correspond to the standard deviation of the corresponding mean values. **h** Log-log plot of the maximum number of particles N_{max} within clusters as a function of the corresponding radius of gyration R_g measured over 1000 frames. The black lines corresponds to lines with slope 1 and 2.

in bulk glass [12, 13]. However, these cooperative moving clusters discussed here reflect the interplay of free surface and bulk glassy properties, which is different from CRRs defined in bulk. As shown in Supplementary Figs. 7a-c, the clusters are typically composed of compact core (red particles) and string-like shell (blue particles), similar to that prevalently observed in bulk glasses [14]. The arrows shown in Supplementary Figs. 7d-f indicate the direction

of particle motion and demonstrate that particles indeed move cooperatively within such clusters. We have also calculated the the mean number of particles within the clusters, $\langle \mathrm{N} \rangle$, as a function of ΔT (Supplementary Fig. 7g). Similar to the thickness of the SGL (l_{SGL}) and penetration depth of fast particles (l_{fast}), $\langle \mathrm{N} \rangle$ exhibits a very comparable dependence on ΔT with a maximum near T_{MCT}. This behavior supports our interpretation that the l_{SGL} and l_{fast} become largest near T_{MCT} where the size of clusters is largest which then facilitates their percolation away from the surface deep towards the bulk. In Supplementary Fig. 7h we have plotted the number of particles within the largest cluster, N_{max}, as a function of its radius of gyration $R_g = (\frac{1}{N}\sum_i (r_i - r_{\mathrm{mean}})^2)^{-\frac{1}{2}}$ where r_i and r_{mean} are the position of particle i and that of the corresponding cluster center of mass. As seen by the power-law dependence, there exists a power-dependence regardless of temperature. In general the radius of gyration of cluster with particle number N should scale as $R_g \sim N^d$ [15] , where the exponent d changes between 1 and 2, depending on whether the clusters are entirely string-like or compact. As shown by the two solid line with slopes one and two, the clusters in our experiments are composed of a mixture of string-like and compact morphology.

Supplementary References

[1] Yoshiyuki Einaga, "Wormlike micelles of polyoxyethylene alkyl ethers CiEj," Polymer journal **41**, 157–173 (2009).

[2] Laurent Helden, Timo Knippenberg, Li Tian, Aubin Archambault, Felix Ginot, and Clemens Bechinger, "Critical Casimir interactions of colloids in micellar critical solutions," Soft Matter **17**, 2737–2741 (2021).

[3] Emanuele Marino, Oleg A. Vasilyev, Bas B. Kluft, Milo J. B. Stroink, Svyatoslav Kondrat, and Peter Schall, "Controlled deposition of nanoparticles with critical Casimir forces," Nanoscale Horiz. **6**, 751–758 (2021).

[4] John C Crocker and David G Grier, "Methods of digital video microscopy for colloidal studies," Journal of colloid and interface science **179**, 298–310 (1996).

[5] C Rycroft, "Voro++: a three-dimensional Voronoi cell library in C++. united states," (2009).

[6] A Gervois, Luc Oger, Patrick Richard, and Jean Paul Troadec, "Voronoi and radical tessella-

tions of packings of spheres," in *International Conference on Computational Science* (Springer, 2002) pp. 95–104.

[7] Kurt Binder and Walter Kob, *Glassy materials and disordered solids: An introduction to their statistical mechanics* (World Scientific, 2011).

[8] Bo Li, Kai Lou, Walter Kob, and Steve Granick, "Anatomy of cage formation in a two-dimensional glass-forming liquid," Nature **587**, 225–229 (2020).

[9] Markus Bayer, Joseph M Brader, Florian Ebert, Matthias Fuchs, E Lange, Georg Maret, Rolf Schilling, Matthias Sperl, and JP Wittmer, "Dynamic glass transition in two dimensions," Physical Review E **76**, 011508 (2007).

[10] W Götze and L Sjögren, "Relaxation processes in supercooled liquids," Reports on progress in Physics **55**, 241 (1992).

[11] Smarajit Karmakar, Chandan Dasgupta, and Srikanth Sastry, "Short-time beta relaxation in glass-forming liquids is cooperative in nature," Physical review letters **116**, 085701 (2016).

[12] Jacob D Stevenson, Jörg Schmalian, and Peter G Wolynes, "The shapes of cooperatively rearranging regions in glass-forming liquids," Nature Physics **2**, 268–274 (2006).

[13] Zexin Zhang, Peter J Yunker, Piotr Habdas, and AG Yodh, "Cooperative rearrangement regions and dynamical heterogeneities in colloidal glasses with attractive versus repulsive interactions," Physical review letters **107**, 208303 (2011).

[14] K Hima Nagamanasa, Shreyas Gokhale, AK Sood, and Rajesh Ganapathy, "Direct measurements of growing amorphous order and non-monotonic dynamic correlations in a colloidal glass-former," Nature Physics **11**, 403–408 (2015).

[15] James Theiler, "Estimating fractal dimension," JOSA A **7**, 1055–1073 (1990).
