# Supplementary material for: Surface melting of a colloidal glass
Source: Nat Commun. 2022 Nov 3;13:6605. doi: 10.1038/s41467-022-34317-2 (PMC9633806; doi:10.1038/s41467-022-34317-2)
Supplement: Supplementary file 2 — Description of Additional Supplementary Files [file 41467_2022_34317_MOESM2_ESM.pdf]

Description of Additional Supplementary Files

Supplementary Movie 1
Surface melting of an equilibrium 2-dimensional colloidal glass at three temperatures (10-x acceleration).

Supplementary Movie 2
Percolation of fast particles (displacement larger than $0.3~\sigma_s$ within $333~{\rm s}$) from surface towards the bulk at three temperatures. The black solid and dashed lines represent z_{sat}^{φ} and $z_{sat}^{\tau_s}$, respectively. The largest cluster percolated from surface is colored in red, other disconnected clusters are filled with other colors (10-x acceleration).

Supplementary Movie 3
Distribution of 10% fastest particles (marked in white) averaged over $333~{\rm s}$ in the region below z_{sat}^{φ}. The white solid and dashed lines represent z_{sat}^{φ} and $z_{sat}^{\tau_s}$, respectively (30-x acceleration).
